# Duration of immune protection of SARS-CoV-2 natural infection against reinfection

**DOI:** 10.1093/jtm/taac109

**Published:** 2022-09-30

**Authors:** Hiam Chemaitelly, Nico Nagelkerke, Houssein H Ayoub, Peter Coyle, Patrick Tang, Hadi M Yassine, Hebah A Al-Khatib, Maria K Smatti, Mohammad R Hasan, Zaina Al-Kanaani, Einas Al-Kuwari, Andrew Jeremijenko, Anvar Hassan Kaleeckal, Ali Nizar Latif, Riyazuddin Mohammad Shaik, Hanan F Abdul-Rahim, Gheyath K Nasrallah, Mohamed Ghaith Al-Kuwari, Adeel A Butt, Hamad Eid Al-Romaihi, Mohamed H Al-Thani, Abdullatif Al-Khal, Roberto Bertollini, Laith J Abu-Raddad

**Affiliations:** Infectious Disease Epidemiology Group, Research Department, Weill Cornell Medicine-Qatar, Cornell University, Doha, Qatar; World Health Organization Collaborating Centre for Disease Epidemiology Analytics on HIV/AIDS, Sexually Transmitted Infections, and Viral Hepatitis, Weill Cornell Medicine–Qatar, Cornell University, Qatar Foundation – Education City, Doha, Qatar; Department of Population Health Sciences, Weill Cornell Medicine, Cornell University, New York, NY, USA; Infectious Disease Epidemiology Group, Research Department, Weill Cornell Medicine-Qatar, Cornell University, Doha, Qatar; Mathematics Program, Department of Mathematics, Statistics, and Physics, College of Arts and Sciences, Qatar University, Doha, Qatar; Hamad Medical Corporation, Doha, Qatar; Biomedical Research Center, QU Health, Qatar University, Doha, Qatar; Wellcome-Wolfson Institute for Experimental Medicine, Queens University, Belfast, UK; Department of Pathology, Sidra Medicine, Doha, Qatar; Biomedical Research Center, QU Health, Qatar University, Doha, Qatar; Department of Biomedical Science, College of Health Sciences, QU Health, Qatar University, Doha, Qatar; Biomedical Research Center, QU Health, Qatar University, Doha, Qatar; Department of Biomedical Science, College of Health Sciences, QU Health, Qatar University, Doha, Qatar; Biomedical Research Center, QU Health, Qatar University, Doha, Qatar; Department of Biomedical Science, College of Health Sciences, QU Health, Qatar University, Doha, Qatar; Department of Pathology, Sidra Medicine, Doha, Qatar; Hamad Medical Corporation, Doha, Qatar; Hamad Medical Corporation, Doha, Qatar; Hamad Medical Corporation, Doha, Qatar; Hamad Medical Corporation, Doha, Qatar; Hamad Medical Corporation, Doha, Qatar; Hamad Medical Corporation, Doha, Qatar; Department of Public Health, College of Health Sciences, QU Health, Qatar University, Doha, Qatar; Biomedical Research Center, QU Health, Qatar University, Doha, Qatar; Department of Biomedical Science, College of Health Sciences, QU Health, Qatar University, Doha, Qatar; Primary Health Care Corporation, Doha, Qatar; Department of Population Health Sciences, Weill Cornell Medicine, Cornell University, New York, NY, USA; Hamad Medical Corporation, Doha, Qatar; Department of Medicine, Weill Cornell Medicine, Cornell University,New York, NY, USA; Ministry of Public Health, Doha, Qatar; Ministry of Public Health, Doha, Qatar; Hamad Medical Corporation, Doha, Qatar; Ministry of Public Health, Doha, Qatar; Infectious Disease Epidemiology Group, Research Department, Weill Cornell Medicine-Qatar, Cornell University, Doha, Qatar; World Health Organization Collaborating Centre for Disease Epidemiology Analytics on HIV/AIDS, Sexually Transmitted Infections, and Viral Hepatitis, Weill Cornell Medicine–Qatar, Cornell University, Qatar Foundation – Education City, Doha, Qatar; Department of Population Health Sciences, Weill Cornell Medicine, Cornell University, New York, NY, USA; Department of Public Health, College of Health Sciences, QU Health, Qatar University, Doha, Qatar

**Keywords:** COVID-19, reinfection, immunity, severe disease, Omicron, cohort study, epidemiology

## Abstract

**Background:**

The future of the severe acute respiratory syndrome coronavirus 2 (SARS-CoV-2) pandemic hinges on virus evolution and duration of immune protection of natural infection against reinfection. We investigated the duration of protection afforded by natural infection, the effect of viral immune evasion on duration of protection and protection against severe reinfection, in Qatar, between 28 February 2020 and 5 June 2022.

**Methods:**

Three national, matched, retrospective cohort studies were conducted to compare the incidence of SARS-CoV-2 infection and coronavirus disease 2019 (COVID-19) severity among unvaccinated persons with a documented SARS-CoV-2 primary infection, to incidence among those infection-naïve and unvaccinated. Associations were estimated using Cox proportional hazard regression models.

**Results:**

Effectiveness of pre-Omicron primary infection against pre-Omicron reinfection was 85.5% [95% confidence interval (CI): 84.8–86.2%]. Effectiveness peaked at 90.5% (95% CI: 88.4–92.3%) in the 7th month after the primary infection, but waned to ~ 70% by the 16th month. Extrapolating this waning trend using a Gompertz curve suggested an effectiveness of 50% in the 22nd month and < 10% by the 32nd month. Effectiveness of pre-Omicron primary infection against Omicron reinfection was 38.1% (95% CI: 36.3–39.8%) and declined with time since primary infection. A Gompertz curve suggested an effectiveness of < 10% by the 15th month. Effectiveness of primary infection against severe, critical or fatal COVID-19 reinfection was 97.3% (95% CI: 94.9–98.6%), irrespective of the variant of primary infection or reinfection, and with no evidence for waning. Similar results were found in sub-group analyses for those ≥50 years of age.

**Conclusions:**

Protection of natural infection against reinfection wanes and may diminish within a few years. Viral immune evasion accelerates this waning. Protection against severe reinfection remains very strong, with no evidence for waning, irrespective of variant, for over 14 months after primary infection.

## Introduction

The future of the severe acute respiratory syndrome coronavirus 2 (SARS-CoV-2) pandemic is uncertain, but it hinges on virus evolution and the level and duration of immune protection of natural infection against reinfection.^1–3^ Although current coronavirus disease 2019 (COVID-19) vaccines had a critical role in reducing COVID-19 hospitalizations and deaths, their rapidly waning immune protection, particularly against the Omicron (B.1.1.529) variant,^4–8^ limits their role in shaping the future of SARS-CoV-2 epidemiology compared with other vaccines, such as vaccinia, which eradicated smallpox.^9^

Seasonal ‘common-cold’ coronaviruses are known to induce by short-term immunity against mild reinfection,^10^ but long-term immunity against severe reinfection.^2^ Although SARS-CoV-2 infection with the original virus or pre-Omicron variants elicited > 80% protection against reinfection with the original virus^11–13^ or with Alpha (B.1.1.7),^14^ Beta (B.1.351)^15^ and Delta (B.1.617.2)^16^ variants, protection against reinfection with Omicron subvariants is below 60%.^16^^,^^17^ Reinfections have become common since Omicron emergence.^17^

We sought to answer three questions of relevance to the future of this pandemic: (i) When infected with a pre-Omicron variant, how long does protection persist against reinfection with pre-Omicron variants? (ii) When infected with a pre-Omicron variant, how long does protection persist against reinfection with an Omicron subvariant? (iii) When infected with any variant, how long does protection persist against severe, critical or fatal COVID-19? Answers to these questions help us to understand duration of protection resulting from natural-infection, effects of viral evasion of the immune system on this duration and effectiveness of natural infection against COVID-19 severity when reinfection occurs.

Three studies were conducted to answer these questions in Qatar, a country that experienced five SARS-CoV-2 waves dominated by each of the original virus,^11^ Alpha,^14^ Beta,^15^ Omicron BA.1 and BA.2,^18^ and currently Omicron BA.4 and BA.5,^19^ in addition to a prolonged low-incidence phase dominated by Delta.^4^

## Methods

### Study population and data sources

This study was conducted in the population of Qatar and analysed COVID-19 data for laboratory testing, vaccination, hospitalization and death, retrieved from the national digital-health information platform. Databases include all SARS-CoV-2-related data, with no missing information since pandemic onset, such as all polymerase chain reaction (PCR) tests, and starting from 5 January 2022, rapid antigen tests conducted at healthcare facilities. Further descriptions of the study population and these national databases were reported previously.^4^^,^^15^^,^^17^^,^^20^^,^^21^

### Study designs and cohorts

We conducted three matched, retrospective cohort studies that emulated randomized ‘target trials’.^21^^,^^22^ In each study, incidence of infection or of severe,^23^ critical^23^ or fatal^24^ COVID-19 was compared in the national cohort of individuals with a documented SARS-CoV-2 primary (first) infection prior to vaccination (designated the primary-infection cohort) to the national (control) cohort of individuals who are infection-naïve and unvaccinated (designated the infection-naïve cohort).

Documentation of infection in all cohorts was based on positive PCR or rapid antigen tests. Laboratory methods are found in Supplementary Section S1. Classification of COVID-19 case severity (acute-care hospitalizations),^23^ criticality (intensive-care-unit hospitalizations) ^23^ and fatality^24^ followed World Health Organization guidelines (Supplementary Section S2).

### Cohort matching and follow-up

Individuals in the primary-infection cohort were exact-matched in a one-to-one ratio by sex, 10-year age group, nationality and comorbidity count (none, 1–2 comorbidities, 3 or more comorbidities) to individuals in the infection-naïve cohort, to control for differences in risk of SARS-CoV-2 infection in Qatar.^20^^,^^25–28^ Matching by these factors was shown previously to provide adequate control of differences in risk of infection.^4^^,^^29–32^ Matching was also done by the calendar week of SARS-CoV-2 testing. That is, an individual who was diagnosed with a primary infection in a specific calendar week was matched to an infection-naïve individual who had a record of a SARS-CoV-2-negative test in that same week (Supplementary Figures S1–S3). This matching ensures that all individuals in all cohorts had active presence in Qatar at the same calendar time. Matching was performed using an iterative process so that each individual in the infection-naïve cohort was alive, infection-free and unvaccinated at the start of follow-up.

SARS-CoV-2 reinfection is conventionally defined as a documented infection ≥90 days after an earlier infection, to avoid misclassification of prolonged PCR positivity as reinfection.^12^^,^^13^^,^^16^ Therefore, each matched pair was followed from the calendar day an individual in the primary-infection cohort completed 90 days after a documented primary infection.

For exchangeability, both members of each matched pair were censored on the date of first-dose vaccination of an individual in either cohort.^21^^,^^33^ Individuals were followed up until the first of any of the following events: a documented SARS-CoV-2 infection, i.e. the first PCR-positive or rapid-antigen-positive test after the start of follow-up, regardless of symptoms, or first-dose vaccination (with matched pair censoring), or death or end of study censoring.

### Pre-Omicron reinfection study

This study estimated the effectiveness of a pre-Omicron primary infection against reinfection with a pre-Omicron variant. Any individual with a documented primary infection between 28 February 2020 (pandemic onset in Qatar) and 30 November 2021 was eligible for inclusion in the primary-infection cohort, provided that the individual received no vaccination before the start of follow-up, 90 days after primary infection. Any individual with a SARS-CoV-2-negative test during this period was eligible for inclusion in the infection-naïve cohort, provided that the individual had no record of infection or vaccination before the start of follow-up. Follow-up was from the 90th day after primary infection until 30 November 2021 (first evidence of Omicron in Qatar^16^^,^^21^ to ensure that incidence during the study was only due to a pre-Omicron variant). The primary study outcome was incidence of infection. The secondary outcome was incidence of severe, critical or fatal COVID-19.

### Omicron reinfection study

This study estimated the effectiveness of a pre-Omicron primary infection against reinfection with an Omicron subvariant. Any individual with a documented primary infection from 28 February 2020 until 30 November 2021 was eligible for inclusion in the primary-infection cohort, absent any record of reinfection or vaccination before the start of follow-up. Any individual with a SARS-CoV-2-negative test during this period was eligible for inclusion in the infection-naïve cohort, absent any record of infection or vaccination before the start of follow-up. Follow-up was from 19 December 2021 (onset of the Omicron wave in Qatar),^16^^,^^21^ if the primary infection occurred ≥90 days before this date. Follow-up was from the 90th day after primary infection if the primary infection occurred < 90 days before 19 December 2021. Follow-up was until 5 June 2022. The primary study outcome was incidence of infection. The secondary outcome was incidence of severe, critical or fatal COVID-19.

### COVID-19 severity reinfection study

This study estimated the effectiveness of a primary infection against severe, critical or fatal COVID-19 reinfection, irrespective of the variant of primary infection or reinfection. Any individual with a documented primary infection between 28 February 2020 and 5 June 2022 was eligible for inclusion in the primary-infection cohort, provided that the individual received no vaccination before the start of follow-up, 90 days after primary infection. Any individual with a SARS-CoV-2-negative test during this period was eligible for inclusion in the infection-naïve cohort, absent any record of infection or vaccination before the start of follow-up. The primary study outcome was incidence of severe, critical or fatal COVID-19. The secondary outcome was incidence of infection.

### Statistical analysis

Eligible and matched cohorts were described using frequency distributions and measures of central tendency, and were compared using standardized mean differences (SMDs). An SMD < 0.1 indicated adequate matching.^34^ Cumulative incidence of infection (defined as the proportion of individuals at risk, whose primary endpoint during follow-up was a reinfection for the primary-infection cohort, or an infection for the infection-naïve cohort) was estimated using the Kaplan–Meier estimator method.^35^ Incidence rate of infection in each cohort, defined as the number of identified infections divided by the number of person-weeks contributed by all individuals in the cohort, was estimated with its 95% confidence interval (CI) using a Poisson log-likelihood regression model with the Stata 17.0 *stptime* command.

The hazard ratio, comparing incidence of infection in both cohorts, and the corresponding 95% CI were calculated using Cox regression adjusted for matching factors with the Stata 17.0 *stcox* command. Schoenfeld residuals and log–log plots for survival curves were used to test the proportional-hazards assumption and to investigate its adequacy. About 95% CIs were not adjusted for multiplicity; thus, they should not be used to infer definitive differences between cohorts. Interactions were not considered. Effectiveness against reinfection was estimated using the equation: Effectiveness = 1−adjusted hazard ratio. Analogous analyses were used when the outcome was severe, critical or fatal COVID-19.

Subgroup analyses were conducted to investigate waning of protection over time. Adjusted hazard ratios were estimated by month since primary infection using separate Cox regressions with ‘failures’ restricted to specific months, in the Pre-Omicron Reinfection Study and the COVID-19 Severity Reinfection Study. Adjusted hazard ratios were also calculated in the Omicron Reinfection Study, but stratified by 3-calendar-month primary-infection sub-cohorts. Additional analyses restricting matched cohorts to those ≥50 years of age were conducted. Sensitivity analyses adjusting effectiveness estimates for differences in testing frequency between cohorts were conducted.

Waning of protection was fitted to the Gompertz function^36^ using the Stata 17.0 *nl* command. This function has been used to describe decay of immunity, such as against smallpox,^36^ and provided a suitable description of waning of protection as assessed by an empiric goodness-of-fit. Statistical analyses were conducted using Stata/SE version 17.0 (Stata Corporation, College Station, TX, USA).

### Oversight

Hamad Medical Corporation and Weill Cornell Medicine-Qatar Institutional Review Boards approved this retrospective study with a waiver of informed consent. The study was reported following the Strengthening the Reporting of Observational Studies in Epidemiology (STROBE) guidelines. The STROBE checklist is found in Supplementary Table S1.

## Results

### Pre-Omicron reinfection study

Supplementary Figure S1 shows the population selection process and Table 1 describes the baseline characteristics of the full and matched cohorts. The matched cohorts each included 290 638 individuals.

**Table 1:** Baseline characteristics of the eligible and matched primary-infection and infection-naïve cohorts in the Pre-Omicron Reinfection Study and the Omicron Reinfection Study

Characteristics	Pre-Omicron Reinfection Study						Omicron Reinfection Study					
	Full eligible cohorts			Matched cohorts^a^			Full eligible cohorts			Matched cohorts^a^		
	Primary-infection cohort	Infection- naïve cohort	SMD^b^	Primary-infection cohort	Infection- naïve cohort	SMD^b^	Primary-infection cohort	Infection- naïve cohort	SMD^b^	Primary-infection cohort	Infection- naïve cohort	SMD^b^
	*N* = 301 943	*N* = 2 329 039		*N* = 290 638	*N* = 290 638		*N* = 127 075	*N* = 2 329 039		*N* = 120 483	*N* = 120 483	
Median age (IQR)—years	32 (24–40)	31 (24–39)	0.05^c^	32 (24–40)	32 (24–40)	0.00^c^	27 (9–36)	31 (24–39)	0.36^c^	27 (9–36)	27 (9–36)	0.00^c^
Age group												
0–9 years	29 583 (9.8)	258 890 (11.1)	0.07	29 022 (10.0)	29 022 (10.0)	0.00	32 465 (25.6)	258 890 (11.1)	0.40	31 834 (26.4)	31 834 (26.4)	0.00
10–19 years	22 830 (7.6)	168 154 (7.2)		21 900 (7.5)	21 900 (7.5)		11 338 (8.9)	168 154 (7.2)		10 630 (8.8)	10 630 (8.8)	
20–29 years	71 904 (23.8)	585 421 (25.1)		70 011 (24.1)	70 011 (24.1)		27 556 (21.7)	585 421 (25.1)		26 467 (22.0)	26 467 (22.0)	
30–39 years	96 468 (32.0)	736 856 (31.6)		93 754 (32.3)	93 754 (32.3)		32 736 (25.8)	736 856 (31.6)		31 340 (26.0)	31 340 (26.0)	
40–49 years	52 822 (17.5)	363 820 (15.6)		50 356 (17.3)	50 356 (17.3)		15 008 (11.8)	363 820 (15.6)		13 908 (11.5)	13 908 (11.5)	
50–59 years	20 772 (6.9)	151 131 (6.5)		19 234 (6.6)	19 234 (6.6)		5453 (4.3)	151 131 (6.5)		4597 (3.8)	4597 (3.8)	
60–69 years	5950 (2.0)	50 033 (2.2)		5127 (1.8)	5127 (1.8)		1937 (1.5)	50 033 (2.2)		1376 (1.1)	1376 (1.1)	
70+ years	1614 (0.5)	14 734 (0.6)		1234 (0.4)	1234 (0.4)		582 (0.5)	14 734 (0.6)		331 (0.3)	331 (0.3)	
Sex												
Male	220 978 (73.2)	1 625 533 (69.8)	0.08	212 685 (73.2)	212 685 (73.2)	0.00	85 134 (67.0)	1 625 533 (69.8)	0.06	80 791 (67.1)	80 791 (67.1)	0.00
Female	80 965 (26.8)	703 506 (30.2)		77 953 (26.8)	77 953 (26.8)		41 941 (33.0)	703 506 (30.2)		39 692 (32.9)	39 692 (32.9)	
Nationality^d^												
Bangladeshi	26 984 (8.9)	152 627 (6.6)	0.24	25 217 (8.7)	25 217 (8.7)	0.00	5808 (4.6)	152 627 (6.6)	0.19	5280 (4.4)	5280 (4.4)	0.00
Egyptian	15 517 (5.1)	121 086 (5.2)		15 275 (5.3)	15 275 (5.3)		7789 (6.1)	121 086 (5.2)		7525 (6.3)	7525 (6.3)	
Filipino	23 569 (7.8)	169 647 (7.3)		23 252 (8.0)	23 252 (8.0)		9244 (7.3)	169 647 (7.3)		9070 (7.5)	9070 (7.5)	
Indian	80 738 (26.7)	641 424 (27.5)		80 426 (27.7)	80 426 (27.7)		33 697 (26.5)	641 424 (27.5)		33 122 (27.5)	33 122 (27.5)	
Nepalese	36 149 (12.0)	201 681 (8.7)		33 226 (11.4)	33 226 (11.4)		11 459 (9.0)	201 681 (8.7)		10 373 (8.6)	10 373 (8.6)	
Pakistani	16 779 (5.6)	126 346 (5.4)		16 182 (5.6)	16 182 (5.6)		7591 (6.0)	126 346 (5.4)		7228 (6.0)	7228 (6.0)	
Qatari	36 177 (12.0)	235 972 (10.1)		36 091 (12.4)	36 091 (12.4)		18 940 (14.9)	235 972 (10.1)		18 813 (15.6)	18 813 (15.6)	
Sri Lankan	9598 (3.2)	59 805 (2.6)		9249 (3.2)	9249 (3.2)		3190 (2.5)	59 805 (2.6)		2950 (2.5)	2950 (2.5)	
Sudanese	8231 (2.7)	55 207 (2.4)		8017 (2.8)	8017 (2.8)		3638 (2.9)	55 207 (2.4)		3430 (2.9)	3430 (2.9)	
Other nationalities^e^	48 201 (16.0)	565 244 (24.3)		43 703 (15.0)	43 703 (15.0)		25 719 (20.2)	565 244 (24.3)		22 692 (18.8)	22 692 (18.8)	
Comorbidity count												
None	241 571 (80.0)	1 991 109 (85.5)	0.15	235 153 (80.9)	235 153 (80.9)	0.00	104 077 (81.9)	1 991 109 (85.5)	0.15	100 533 (83.4)	100 533 (83.4)	0.00
1–2	48 776 (16.2)	268 390 (11.5)		45 342 (15.6)	45 342 (15.6)		20 547 (16.2)	268 390 (11.5)		18 271 (15.2)	18 271 (15.2)	
3+	11 596 (3.8)	69 540 (3.0)		10 143 (3.5)	10 143 (3.5)		2451 (1.9)	69 540 (3.0)		1679 (1.4)	1679 (1.4)	

IQR denotes interquartile range and SMD standardized mean difference.

a Individuals with a documented primary SARS-CoV-2 infection were exact-matched in a 1:1 ratio by sex, 10-year age group, nationality, comorbidity count and calendar week of the SARS-CoV-2 test to the first eligible infection-naïve individual.

b SMD is the difference in the mean of a covariate between groups divided by the pooled standard deviation. An SMD < 0.1 indicates adequate matching.

c SMD is for the mean difference between groups divided by the pooled standard deviation.

d Nationalities were chosen to represent the most populous groups in Qatar.

e These comprise 150 other nationalities in the unmatched primary-infection cohort and 183 other nationalities in the unmatched infection-naïve cohort, and 133 other nationalities in the matched primary-infection cohort and 133 other nationalities in the matched infection-naïve cohort in the Pre-Omicron Reinfection Study. These also comprise 145 other nationalities in the unmatched primary-infection cohort and 183 other nationalities in the unmatched infection-naïve cohort, and 123 other nationalities in the matched primary-infection cohort and 123 other nationalities in the matched infection-naïve cohort in the Omicron Reinfection Study.

**Figure 1:**
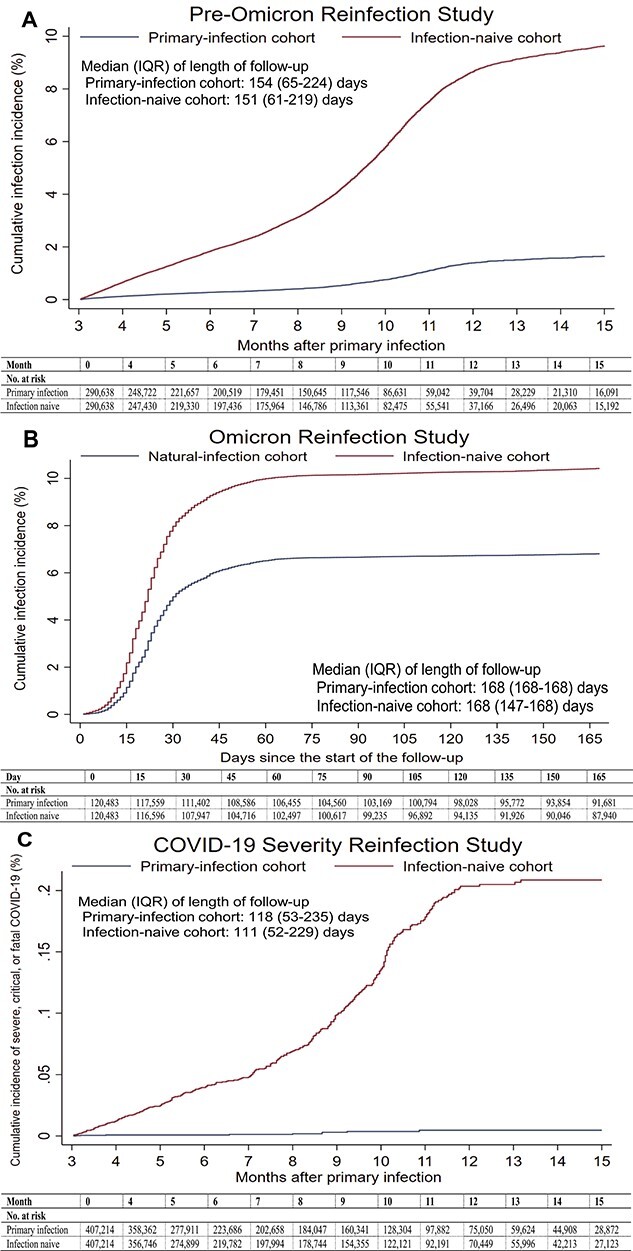
(A) Cumulative incidence of infection in the pre-Omicron Reinfection Study. (B) Cumulative incidence of infection in the Omicron Reinfection Study. (C) Cumulative incidence of severe, critical or fatal COVID-19 in the COVID-19 Severity Reinfection Study.

There were 1806 reinfections in the primary-infection cohort during follow-up, of which six progressed to severe and one to fatal COVID-19 (Supplementary Figure S1). Meanwhile, there were 11 957 infections in the infection-naïve cohort, of which 297 progressed to severe, 19 to critical and 12 to fatal COVID-19. Cumulative incidence of infection was 1.7% (95% CI: 1.6–1.8%) for the primary-infection cohort and 9.6% (95% CI: 9.4–9.9%) for the infection-naïve cohort, 15 months after the primary infection (Figure 1A).

**Figure 2:**
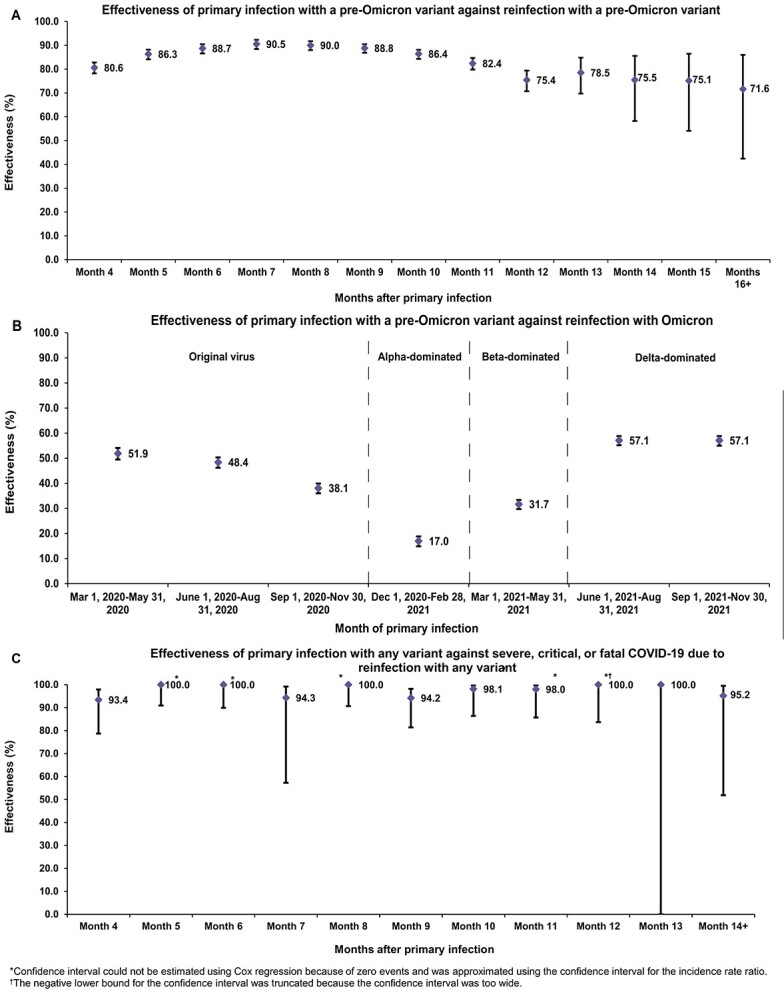
(A) Effectiveness of pre-Omicron primary infection against pre-Omicron reinfection. (B) Effectiveness of pre-Omicron primary infection against Omicron reinfection. (C) Effectiveness of primary infection with any variant against severe, critical or fatal COVID-19 due to reinfection with any variant.

**Figure 3:**
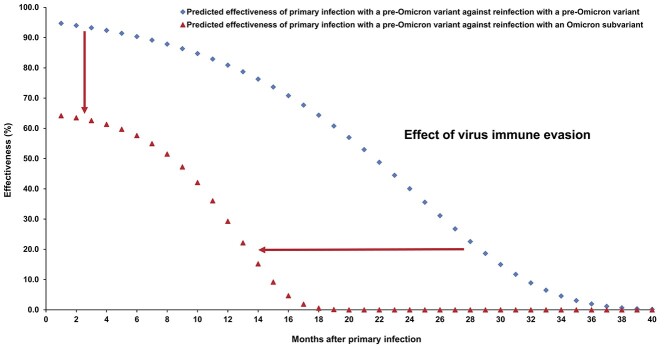
Extrapolated effectiveness of pre-Omicron primary infection against pre-Omicron reinfection, and extrapolated effectiveness of pre-Omicron primary infection against Omicron reinfection.

The overall hazard ratio for infection, adjusted for sex, 10-year age group, 10 nationality groups, comorbidity count and SARS-CoV-2 test calendar week, was 0.14 (95% CI: 0.14–0.15; Table 2). Effectiveness of pre-Omicron primary infection against pre-Omicron reinfection was 85.5% (95% CI: 84.8–86.2%). Effectiveness increased slowly after the primary infection and reached 90.5% (95% CI: 88.4–92.3%) in the seventh month after the primary infection (Figure 2A). Starting in the 8th month, effectiveness waned slowly and reached ~ 70% by the 16th month. Fitting the waning of protection to a Gompertz curve suggested that effectiveness reaches 50% in the 22nd month and < 10% by the 32nd month (Figure 3).

**Table 2:** Hazard ratios for the incidence of SARS-CoV-2 infection and incidence of severe, critical or fatal COVID-19 in the Pre-Omicron Reinfection Study, Omicron Reinfection Study and COVID-19 Severity Reinfection Study

Epidemiological measure	Primary-infection cohort	Infection-naïve cohort
Pre-Omicron Reinfection Study		
Primary outcome		
Total follow-up time (person-weeks)	6 578 466	6 432 430
Incidence rate of infection (per 10 000 person-weeks)	2.8 (2.6–2.9)	18.6 (18.3–18.9)
Unadjusted hazard ratio for SARS-CoV-2 infection (95% CI)	0.15 (0.14–0.15)	
Adjusted hazard ratio for SARS-CoV-2 infection^a^ (95% CI)	0.14 (0.14–0.15)	
Effectiveness against SARS-CoV-2 infection in % (95% CI)	85.5 (84.8–86.2)	
Secondary outcome		
Unadjusted hazard ratio for severe, critical or fatal COVID-19^b^ (95% CI)	0.02 (0.01–0.04)	
Adjusted hazard ratio for severe, critical or fatal COVID-19^a,b^ (95% CI)	0.02 (0.01–0.04)	
Effectiveness against severe, critical or fatal COVID-19^a,b^ (95% CI)	98.0 (95.7–99.0)	
Omicron Reinfection Study		
Primary outcome		
Total follow-up time (person-weeks)	2 493 870	2 411 571
Incidence rate of infection (per 10 000 person-weeks)	32.1 (31.4–32.8)	50.7 (49.8–51.6)
Unadjusted hazard ratio for SARS-CoV-2 infection (95% CI)	0.64 (0.62–0.66)	
Adjusted hazard ratio for SARS-CoV-2 infection^a^ (95% CI)	0.62 (0.60–0.64)	
Effectiveness against SARS-CoV-2 infection in % (95% CI)	38.1 (36.3–39.8)	
Secondary outcome		
Unadjusted hazard ratio for severe, critical or fatal COVID-19^b^ (95% CI)	0.13 (0.05–0.33)	
Adjusted hazard ratio for severe, critical or fatal COVID-19^a,b^ (95% CI)	0.11 (0.04–0.29)	
Effectiveness against severe, critical or fatal COVID-19^a,b^ (95% CI)	88.6 (70.9–95.5)	
Reinfection COVID-19 Severity Study		
Primary outcome		
Total follow-up time (person-weeks)	9 290 507	9 022 235
Incidence rate of severe, critical or fatal COVID-19 (per 10 000 person-weeks)	0.01 (0.01–0.02)	0.40 (0.36–0.44)
Unadjusted hazard ratio for severe, critical or fatal COVID-19^b^ (95% CI)	0.03 (0.01–0.05)	
Adjusted hazard ratio for severe, critical or fatal COVID-19^a,b^ (95% CI)	0.03 (0.01–0.05)	
Effectiveness against severe, critical or fatal COVID-19^a,b^ (95% CI)	97.3 (94.9–98.6)	
Secondary outcome		
Unadjusted hazard ratio for SARS-CoV-2 infection (95% CI)	0.31 (0.30–0.32)	
Adjusted hazard ratio for SARS-CoV-2 infection^a^ (95% CI)	0.31 (0.30–0.31)	
Effectiveness against SARS-CoV-2 infection in % (95% CI)	69.4 (68.6–70.3)	

CI denotes confidence interval, COVID-19 coronavirus disease 2019, and SARS-CoV-2 severe acute respiratory syndrome coronavirus 2.

a Cox regression analysis adjusted for sex, 10-year age group (Table 1), 10 nationality groups (Table 1), comorbidity count (Table 1) and calendar week of the SARS-CoV-2 test.

b Severity,^23^ criticality^23^ and fatality^24^ were defined according to the World Health Organization guidelines.

Effectiveness of pre-Omicron primary infection against severe, critical or fatal COVID-19 due to pre-Omicron reinfection was 98.0% (95% CI: 95.7–99.0%; Table 2). In the additional analysis restricting the matched cohorts to the sub-cohorts ≥50 years of age (25 595 individuals), effectiveness against reinfection and against severe, critical or fatal COVID-19 reinfection was 90.7% (95% CI: 88.4–92.5%) and 97.4% (95% CI: 91.9–99.2%), respectively. In the sensitivity analysis adjusting the overall hazard ratio by the ratio of testing frequency, effectiveness against reinfection was 79.5% (95% CI: 78.4–80.5%). More results are in Section S3.

### Omicron reinfection study

Supplementary Figure S2 shows the process of population selection and Table 1 describes the baseline characteristics of the full and matched cohorts. The matched cohorts each included 120 483 individuals.

There were 7995 reinfections in the primary-infection cohort during follow-up, of which five progressed to severe COVID-19 (Supplementary Figure S2). Meanwhile, there were 12 230 infections in the infection-naïve cohort, of which 26 progressed to severe, 7 to critical and 5 to fatal COVID-19. Cumulative incidence of infection was 6.8% (95% CI: 6.7–6.9%) for the primary-infection cohort and 10.4% (95% CI: 10.2–10.6%) for the infection-naïve cohort, 165 days after the start of follow-up (Figure 1B).

The overall adjusted hazard ratio for infection was 0.62 (95% CI: 0.60–0.64; Table 2). Effectiveness of pre-Omicron primary infection against Omicron reinfection was 38.1% (95% CI: 36.3–39.8%). Effectiveness varied for the primary-infection sub-cohorts (Figure 2B). It was ~ 60% for those with a more recent primary infection, between 1 June 2021 and 30 November 2021, during Delta-dominated incidence.^4^^,^^37^^,^^38^ Effectiveness declined with time since primary infection and was 17.0% (95% CI: 10.1–23.5%) for those with a primary infection between 1 December 2020 and 28 February 2021, during Alpha-dominated incidence.^4^^,^^37^^,^^38^ However, higher effectiveness of ~ 50% was estimated for those with a primary infection before 31 August 2020, during original-virus incidence (note discussion in Supplementary Section S3).^4^^,^^37^^,^^38^ Fitting the waning of protection to a Gompertz curve suggested that effectiveness reaches 50% in the 8th month after primary infection and < 10% by the 15th month (Figure 3).

Effectiveness of pre-Omicron primary infection against severe, critical or fatal COVID-19 due to Omicron reinfection was 88.6% (95% CI: 70.9–95.5%; Table 2). In the additional analysis restricting the matched cohorts to the sub-cohorts ≥50 years of age (6304 individuals), effectiveness against reinfection and against severe, critical or fatal COVID-19 reinfection was 21.6% (95% CI: 11.1–31.0%) and 84.6% (95% CI: 59.7–94.1%), respectively. In the sensitivity analysis adjusting the overall hazard ratio by the ratio of testing frequency, effectiveness against reinfection was 31.7% (95% CI: 29.7–33.6%). More results are in Supplementary Section S3.

### COVID-19 severity reinfection study

Supplementary Figure S3 shows the process of population selection and Supplementary Table S2 describes the baseline characteristics of the full and matched cohorts. The matched cohorts each included 407 214 individuals.

There were 7082 reinfections in the primary-infection cohort during follow-up, of which nine progressed to severe and one progressed to fatal COVID-19 (Supplementary Figure S3). Meanwhile, there were 21 645 infections in the infection-naïve cohort, of which 315 progressed to severe, 25 to critical and 18 to fatal COVID-19. Cumulative incidence of severe, critical or fatal COVID-19 was 0.00% (95% CI: 0.00–0.01%) for the primary-infection cohort and 0.21% (95% CI: 0.19–0.23%) for the infection-naïve cohort, 15 months after the primary infection (Figure 1C).

The overall adjusted hazard ratio for severe, critical or fatal COVID-19 was estimated at 0.03 (95% CI: 0.01–0.05; Table 2). Effectiveness of primary infection with any variant against severe, critical or fatal COVID-19 due to reinfection with any variant was 97.3% (95% CI: 94.9–98.6%). Variation by month after primary infection was negligible, with no evidence for waning (Figure 2C). Effectiveness was ~ 100% up to the 14th month after primary infection.

Effectiveness of primary infection with any variant against reinfection with any variant was 69.4% (95% CI: 68.6–70.3%; Table 2). In the additional analysis restricting the matched cohorts to the sub-cohorts ≥50 years of age (31 108 individuals), effectiveness against reinfection and against severe, critical or fatal COVID-19 was 75.3% (95% CI: 72.0–78.2%) and 95.4% (95% CI: 89.4–98.0%), respectively. More results are in Supplementary Section S3.

## Discussion

Protection of natural infection waned with time after primary infection, prior to Omicron emergence, and reached ~ 70% by the 16th month. This waning likely reflects genuine waning in biological immunity rather than viral immune evasion, as pre-Omicron variants demonstrated much less immune evasion than Omicron.^14–17^ This waning in natural immunity mirrors that of vaccine immunity,^4^^,^^6^^,^^31^ but at a slower rate. Vaccine immunity may last for only a year,^4^^,^^6^^,^^31^ but natural immunity, assuming Gompertz decay, may last for 3 years, as also suggested by long-term follow-up of SARS-CoV-1-associated antibodies,^39^ and incidentally not dissimilar to pandemic-influenza-associated antibodies.^40^

Immune evasion of Omicron subvariants reduced overall protection of pre-Omicron natural immunity and accelerated its waning (Figure 3), mirroring the effect of Omicron on vaccine immunity, but at a slower rate. Vaccine immunity against Omicron subvariants lasts for < 6 months,^5^^,^^7^^,^^8^^,^^41^ but pre-Omicron natural immunity, assuming Gompertz decay, may last for just over a year.

Despite waning protection against reinfection, strikingly, there was no evidence for waning of protection against severe COVID-19 at reinfection. This remained ~ 100%, even 14 months after the primary infection, with no appreciable effect for Omicron immune evasion in reducing it. This pattern also mirrors that of vaccine immunity, which wanes rapidly against infection, but is durable against severe COVID-19, regardless of variant.^4^^,^^6–8^^,^^31^

Infection with common-cold coronaviruses, and perhaps influenza,^42^ induces only a year-long immunity against reinfection,^10^ but life-long immunity against severe reinfection.^2^ To what extent this pattern reflects waning in biological immunity or immune evasion with virus evolution over the global season is unclear. The above results suggest that SARS-CoV-2 may exhibit a similar pattern to that of common-cold coronaviruses within few years. Short-term biological immunity against reinfection of 3 years may decline as a result of viral evolution and immune evasion, leading to periodic (possibly annual) waves of infection. However, the lasting immunity against severe reinfection will contribute to a pattern of benign infection. Most primary infections would occur in childhood and would likely not be severe. Adults would only experience periodic reinfections, also not likely to be severe.

This study has limitations. We investigated the incidence of documented infections, but other infections may have occurred and gone undocumented. Undocumented infections confer immunity or boost existing immunity,^43–46^ thereby perhaps affecting the estimates (note Supplementary Section S3). Differences in testing frequency existed between the followed cohorts, but these were small and adjusted estimates in sensitivity analyses confirmed similar findings. Gompertz function^36^ was used to parametrize immunity decay, based on empiric goodness-of-fit, but this analysis serves only as an informed exploratory extrapolation that remains to be confirmed with more follow-up of cohorts. With Qatar’s young population, our findings may not be generalizable to other countries where elderly citizens constitute a larger proportion of the total population. However, additional analyses restricting the matched cohorts to those ≥50 years of age showed findings resembling those for the total population.

Depletion of the primary-infection cohorts by COVID-19 mortality at time of primary infection may have biased these cohorts toward healthier individuals with stronger immune responses. However, COVID-19 mortality has been low in Qatar’s predominantly young population,^20^^,^^47^ totaling 679 COVID-19 deaths (<0.1% of primary infections) up to 29 June 2022, and much smaller than the size of the study cohorts. A survival effect seems unlikely to explain or appreciably affect the study findings, apart perhaps from protection against severe COVID-19. The present studies investigated the protection of any documented infection regardless of presence of symptoms or severe disease, but it is conceivable that there could be differences in the protection depending on the severity scale of symptoms at the primary infection. Such differences in protection remains to be investigated.

Vaccination prior to Omicron introduction in Qatar was effective in preventing infection acquisition.^4^^,^^21^^,^^30^^,^^31^^,^^48^ All three pre-Omicron waves in this country occurred before the mass scale-up of vaccination.^4^ The subsequent rapid scale-up of vaccination led to large attrition in the followed cohorts due to censoring at vaccination (Supplementary Figure S1). Vaccine rollout proceeded in phases in which vaccination was prioritized first to frontline healthcare workers, persons with severe or multiple chronic conditions and persons ≥70 years of age.^4^ Vaccination was then gradually extended by one age group at a time and to select professional groups (such as teachers), with age being a principal criterion for vaccine eligibility throughout the rollout.^4^ Vaccination of children and adolescents was substantially delayed compared with that of adults.^41^ Therefore, there are differences between vaccinated individuals and unvaccinated individuals and these differences changed with time. These factors indirectly affected the composition of the primary-infection cohorts and their matched cohorts. The cohorts thus may not be exactly representative of the total population of Qatar. The reported results are applicable to the specific cohorts of the present studies and may not be generalizable to other populations.

As an observational study, investigated cohorts were neither blinded nor randomized, so unmeasured or uncontrolled confounding cannot be excluded. Although matching was done for sex, age, nationality, comorbidity count and timing of primary infection, this was not possible for other factors such as geography or occupation, as such data were unavailable. However, Qatar is essentially a city state and infection incidence was broadly distributed across neighbourhoods. Nearly 90% of Qatar’s population are expatriates from over 150 countries, coming here because of employment.^20^ Most are craft and manual workers working in development projects.^20^ Nationality, age and sex provide a powerful proxy for socio-economic status in this country.^20^^,^^25–28^ Nationality alone is strongly associated with occupation.^20^^,^^26–28^

Matching was done to control for factors that affect infection exposure in Qatar.^20^^,^^25–28^ The matching prescription had already been investigated in previous studies of different epidemiologic designs, and using control groups to test for null effects.^4^^,^^29–32^ These control groups included unvaccinated cohorts versus vaccinated cohorts within 2 weeks of the first dose,^4^^,^^29–31^ when vaccine protection is negligible,^49^ and mRNA-1273 versus BNT162b2-vaccinated cohorts, also in the first 2 weeks after the first dose.^32^ These studies have shown that this prescription provides adequate control of the differences in infection exposure.^4^^,^^29–32^ These analyses were implemented using Qatar’s total population with large sample sizes, thus minimizing the likelihood of bias.

In conclusion, protection of natural infection against reinfection wanes and may diminish within a few years. Omicron immune evasion accelerates this waning. Meanwhile, protection against severe reinfection is very strong with no evidence for waning, regardless of variant, for over 14 months after the primary infection.

## Author contributions

H.C. co-designed the study, performed the statistical analyses and co-wrote the first draft of the article. L.J.A. conceived and co-designed the study, led the statistical analyses and co-wrote the first draft of the article. N.G. provided technical advice and insights about immunity decay. P.T. and M.R.H. conducted multiplex, RT-qPCR variant screening and viral genome sequencing. H.Y., H.A.K. and M.S. conducted viral genome sequencing. All authors contributed to data collection and acquisition, database development, discussion and interpretation of the results, and to the writing of the manuscript. All authors have read and approved the final manuscript.

## Competing interests

Dr Butt has received institutional grant funding from Gilead Sciences unrelated to the work presented in this paper. Otherwise, we declare no competing interests.

## Supplementary Material

Supplementary_Appendix_taac109
