# Supplementary material for: Duration of immune protection of SARS-CoV-2 natural infection against reinfection
Source: J Travel Med. 2022 Sep 30;29(8):taac109. doi: 10.1093/jtm/taac109 (PMC9619565; doi:10.1093/jtm/taac109)
Supplement: Supplementary_Appendix_taac109 [file supplementary_appendix_taac109.pdf]

Supplementary Appendix

Table of Contents

Section S1. Laboratory methods and variant ascertainment 2

Real-time reverse-transcription polymerase chain reaction testing 2

Rapid antigen testing 2

Classification of infections by variant type 3

Section S2. COVID-19 severity, criticality, and fatality classification 4

Section S3. Additional material for the Results section 6

Table S1. STROBE checklist for cohort studies 8

Figure S1. Cohort selection for investigating immune protection of primary infection with a pre-Omicron variant against re-infection with a pre-Omicron variant (Pre-Omicron Reinfection Study). 10

Figure S2. Cohort selection for investigating immune protection of primary infection with a pre-Omicron variant against re-infection with Omicron subvariants (Omicron Reinfection Study). 11

Figure S3. Cohort selection for investigating immune protection of primary infection with any variant against severe, critical, or fatal COVID-19 due to reinfection with any variant. 12

Table S2. Baseline characteristics of the eligible and matched primary-infection and infection-naive cohorts in the COVID-19 Severity Reinfection Study. 13

References 14

Section S1. Laboratory methods and variant ascertainment

Real-time reverse-transcription polymerase chain reaction testing

Nasopharyngeal and/or oropharyngeal swabs were collected for polymerase chain reaction (PCR) testing and placed in Universal Transport Medium (UTM). Aliquots of UTM were: 1) extracted on KingFisher Flex (Thermo Fisher Scientific, USA), MGISP-960 (MGI, China), or ExiPrep 96 Lite (Bioneer, South Korea) followed by testing with real-time reverse-transcription PCR (RT-qPCR) using TaqPath COVID-19 Combo Kits (Thermo Fisher Scientific, USA) on an ABI 7500 FAST (Thermo Fisher Scientific, USA); 2) tested directly on the Cepheid GeneXpert system using the Xpert Xpress SARS-CoV-2 (Cepheid, USA); or 3) loaded directly into a Roche cobas 6800 system and assayed with the cobas SARS-CoV-2 Test (Roche, Switzerland). The first assay targets the viral S, N, and ORF1ab gene regions. The second targets the viral N and E-gene regions, and the third targets the ORF1ab and E-gene regions.

All PCR testing was conducted at the Hamad Medical Corporation Central Laboratory or Sidra Medicine Laboratory, following standardized protocols.

Rapid antigen testing

Severe acute respiratory syndrome coronavirus 2 (SARS-CoV-2) antigen tests were performed on nasopharyngeal swabs using one of the following lateral flow antigen tests: Panbio COVID-19 Ag Rapid Test Device (Abbott, USA); SARS-CoV-2 Rapid Antigen Test (Roche, Switzerland); Standard Q COVID-19 Antigen Test (SD Biosensor, Korea); or CareStart COVID-19 Antigen Test (Access Bio, USA). All antigen tests were performed point-of-care according to each manufacturer's instructions at public or private hospitals and clinics throughout Qatar with prior authorization and training by the Ministry of Public Health (MOPH). Antigen test results

were electronically reported to the MOPH in real time using the Antigen Test Management System which is integrated with the national Coronavirus Disease 2019 (COVID-19) database.

Classification of infections by variant type

Surveillance for SARS-CoV-2 variants in Qatar is based on viral genome sequencing and multiplex RT-qPCR variant screening[1] of random positive clinical samples,[2-7] complemented by deep sequencing of wastewater samples.[4,8] Further details on the viral genome sequencing and multiplex RT-qPCR variant screening throughout the SARS-CoV-2 waves in Qatar can be found in previous publications.[2-7,9-14]

Section S2. COVID-19 severity, criticality, and fatality classification

Classification of COVID-19 case severity (acute-care hospitalizations),[15] criticality (intensive-care-unit hospitalizations),[15] and fatality[16] followed World Health Organization (WHO) guidelines. Assessments were made by trained medical personnel independent of study investigators and using individual chart reviews, as part of a national protocol applied to every hospitalized COVID-19 patient. Each hospitalized COVID-19 patient underwent an infection severity assessment every three days until discharge or death. We classified individuals who progressed to severe, critical, or fatal COVID-19 between the time of the documented infection and the end of the study based on their worst outcome, starting with death,[16] followed by critical disease,[15] and then severe disease.[15]

Severe COVID-19 disease was defined per WHO classification as a SARS-CoV-2 infected person with "oxygen saturation of <90% on room air, and/or respiratory rate of >30 breaths/minute in adults and children >5 years old (or ≥60 breaths/minute in children <2 months old or ≥50 breaths/minute in children 2-11 months old or ≥40 breaths/minute in children 1–5 years old), and/or signs of severe respiratory distress (accessory muscle use and inability to complete full sentences, and, in children, very severe chest wall indrawing, grunting, central cyanosis, or presence of any other general danger signs)".[15] Detailed WHO criteria for classifying SARS-CoV-2 infection severity can be found in the WHO technical report.[15]

Critical COVID-19 disease was defined per WHO classification as a SARS-CoV-2 infected person with "acute respiratory distress syndrome, sepsis, septic shock, or other conditions that would normally require the provision of life sustaining therapies such as mechanical ventilation (invasive or non-invasive) or vasopressor therapy".[15] Detailed WHO criteria for classifying SARS-CoV-2 infection criticality can be found in the WHO technical report.[15]

COVID-19 death was defined per WHO classification as “a death resulting from a clinically compatible illness, in a probable or confirmed COVID-19 case, unless there is a clear alternative cause of death that cannot be related to COVID-19 disease (e.g. trauma). There should be no period of complete recovery from COVID-19 between illness and death. A death due to COVID-19 may not be attributed to another disease (e.g. cancer) and should be counted independently of preexisting conditions that are suspected of triggering a severe course of COVID-19”. Detailed WHO criteria for classifying COVID-19 death can be found in the WHO technical report.[16]

Section S3. Additional material for the Results section

Pre-Omicron Reinfection Study

The median time of follow-up was 154 days (interquartile range (IQR), 65-224 days) for the primary-infection cohort and 151 days (IQR, 61-219 days) for the infection-naïve cohort (Figure 1A). The proportion of individuals who had a SARS-CoV-2 test during follow-up was 29.7% for the primary-infection cohort and 36.4% for the infection-naïve cohort. The testing frequency was 0.56 and 0.79 tests per person, respectively.

The pattern of waning of protection in Figure 2A was fitted to a Gompertz function,[17] but after setting the effectiveness values in months 4-6 after primary infection at 90.5%, the value at the 7th month. This was done to correct for the likely underestimation of the observed effectiveness in these months because of the effect of PCR-positive tests that reflected prolonged infections as opposed to true reinfections.[18-23] The fitted Gompertz function suggested that effectiveness against reinfection reaches 50% in the 22nd month after primary infection, and reaches <10% by the 32nd month (Figure 3).

Omicron Reinfection Study

The median time of follow-up was 168 days (IQR, 168-168 days) for the primary-infection cohort and 168 days (IQR, 147-168 days) for the infection-naïve cohort (Figure 1B). The proportion of individuals who had a SARS-CoV-2 test during follow-up was 29.9% for the primary-infection cohort and 32.5% for the infection-naïve cohort. The testing frequency was 0.50 and 0.55 tests per person, respectively.

The pattern of waning of protection in Figure 2B was fitted to a Gompertz function,[17] but after excluding the effectiveness values before December 1, 2020. Incidence before this date, that is

during the first wave, affected mainly the craft and manual workers population of Qatar, that constitutes 60% of the total population and where SARS-CoV-2 seroprevalence exceeded 50% by the end of the first wave.[24-28] This population segment has also the lowest testing rates in the population.[29] It is possible that many of these workers may have had mild or asymptomatic reinfections[30,31] that were not documented during the four subsequent waves.[11,12,32,33] This undocumented immune boosting, subsequent to the primary infection, may explain the higher-than-expected effectiveness for those who had their primary infection prior to December 1, 2020. The fitted Gompertz function suggested that effectiveness against reinfection reaches 50% in the 8th month after primary infection, and reaches <10% by the 15th month (Figure 3).

COVID-19 Severity Reinfection Study

The median time of follow-up was 118 days (IQR, 53-235 days) for the primary-infection cohort and 111 days (IQR, 52-229 days) for the infection-naïve cohort (Figure 1C). The proportion of individuals who had a SARS-CoV-2 test during follow-up was 28.0% for the primary-infection cohort and 31.7% for the infection-naïve cohort. The testing frequency was 0.56 and 0.70 tests per person, respectively.

Since there was no evidence for waning in effectiveness against severe, critical, or fatal COVID-19 due to reinfection, there was no relevance to fit a Gompertz function to the effectiveness trend after primary infection.

Table S1. STROBE checklist for cohort studies.

	Item No	Recommendation	Main Text page
**Title and abstract**	1	(*a*) Indicate the study's design with a commonly used term in the title or the abstract	Abstract
		(*b*) Provide in the abstract an informative and balanced summary of what was done and what was found	Abstract
**Introduction**			
Background/rationale	2	Explain the scientific background and rationale for the investigation being reported	Introduction
Objectives	3	State specific objectives, including any prespecified hypotheses	Introduction
**Methods**			
Study design	4	Present key elements of study design early in the paper	Methods ('Study designs and cohorts')
Setting	5	Describe the setting, locations, and relevant dates, including periods of recruitment, exposure, follow-up, and data collection	Methods ('Study designs and cohorts', 'Cohort matching and follow-up', 'Pre-Omicron Reinfection Study', 'Omicron Reinfection Study', & 'COVID-19 Severity Reinfection Study') & Figures S1-S3 in Supplementary Appendix
Participants	6	(*a*) Give the eligibility criteria, and the sources and methods of selection of participants. Describe methods of follow-up<br>(*b*) For matched studies, give matching criteria and number of exposed and unexposed	Methods ('Study designs and cohorts', 'Cohort matching and follow-up', 'Pre-Omicron Reinfection Study', 'Omicron Reinfection Study', & 'COVID-19 Severity Reinfection Study') & Figures S1-S3 in Supplementary Appendix
Variables	7	Clearly define all outcomes, exposures, predictors, potential confounders, and effect modifiers. Give diagnostic criteria, if applicable	Methods ('Study designs and cohorts', 'Cohort matching and follow-up', 'Pre-Omicron Reinfection Study', 'Omicron Reinfection Study', & 'COVID-19 Severity Reinfection Study'), Table 1, & Table S2 & Sections S1& S2 in Supplementary Appendix
Data sources/ measurement	8*	For each variable of interest, give sources of data and details of methods of assessment (measurement). Describe comparability of assessment methods if there is more than one group	Methods ('Study population and data sources' & 'Statistical analysis'), Table 1, & Table S2 & Sections S1 & S2 in Supplementary Appendix
Bias	9	Describe any efforts to address potential sources of bias	Methods ('Cohort matching and follow-up')
Study size	10	Explain how the study size was arrived at	Figures S1-S3 in Supplementary Appendix
Quantitative variables	11	Explain how quantitative variables were handled in the analyses. If applicable, describe which groupings were chosen and why	Methods ('Cohort matching and follow-up') & Table 1 & Table S2 in Supplementary Appendix
Statistical methods	12	(*a*) Describe all statistical methods, including those used to control for confounding	Methods ('Statistical analysis')
		(*b*) Describe any methods used to examine subgroups and interactions	Methods ('Statistical analysis', paragraph 3)
		(*c*) Explain how missing data were addressed	Not applicable, see Methods ('Study population and data sources')
		(*d*) If applicable, explain how loss to follow-up was addressed	Not applicable, see Methods ('Study designs and cohorts', paragraph 1)
		(*e*) Describe any sensitivity analyses	Methods ('Statistical analysis', paragraph 3)
**Results**			
Participants	13*	(a) Report numbers of individuals at each stage of study—eg numbers potentially eligible, examined for eligibility, confirmed eligible, included in the study, completing follow-up, and analysed<br>(b) Give reasons for non-participation at each stage<br>(c) Consider use of a flow diagram	Figures S1-S3 in Supplementary Appendix
Descriptive data	14	(a) Give characteristics of study participants (eg demographic, clinical, social) and information on exposures and potential confounders	Table 1 & Table S2 in Supplementary Appendix
		(b) Indicate number of participants with missing data for each variable of interest	Not applicable, see Methods ('Study population and data sources')

		(c) Summarise follow-up time (eg, average and total amount)	Results (‘Pre-Omicron Reinfection Study’, ‘Omicron Reinfection Study’, & ‘COVID-19 Severity Reinfection Study’), Figure 1, & Table 2
Outcome data	15	Report numbers of outcome events or summary measures over time	Results (‘Pre-Omicron Reinfection Study’, ‘Omicron Reinfection Study’, & ‘COVID-19 Severity Reinfection Study’), Figure 1, & Table 2
Main results	16	(a) Give unadjusted estimates and, if applicable, confounder-adjusted estimates and their precision (eg, 95% confidence interval). Make clear which confounders were adjusted for and why they were included	Results (‘Pre-Omicron Reinfection Study’, ‘Omicron Reinfection Study’, & ‘COVID-19 Severity Reinfection Study’), & Table 2
		(b) Report category boundaries when continuous variables were categorized	Table 1 & Table S2 in Supplementary Appendix
		(c) If relevant, consider translating estimates of relative risk into absolute risk for a meaningful time period	Not applicable
Other analyses	17	Report other analyses done—eg analyses of subgroups and interactions, and sensitivity analyses	Results (‘Pre-Omicron Reinfection Study’, ‘Omicron Reinfection Study’, & ‘COVID-19 Severity Reinfection Study’) & Figures 2-3
Discussion			
Key results	18	Summarise key results with reference to study objectives	Discussion, paragraphs 1-4
Limitations	19	Discuss limitations of the study, taking into account sources of potential bias or imprecision. Discuss both direction and magnitude of any potential bias	Discussion, paragraphs 5-9
Interpretation	20	Give a cautious overall interpretation of results considering objectives, limitations, multiplicity of analyses, results from similar studies, and other relevant evidence	Discussion, paragraph 10
Generalisability	21	Discuss the generalisability (external validity) of the study results	Discussion, paragraphs 7-8
Other information			
Funding	22	Give the source of funding and the role of the funders for the present study and, if applicable, for the original study on which the present article is based	Sources of support & acknowledgements

Figure S1. Cohort selection for investigating immune protection of primary infection with a pre-Omicron variant against re-infection with a pre-Omicron variant (Pre-Omicron Reinfection Study).

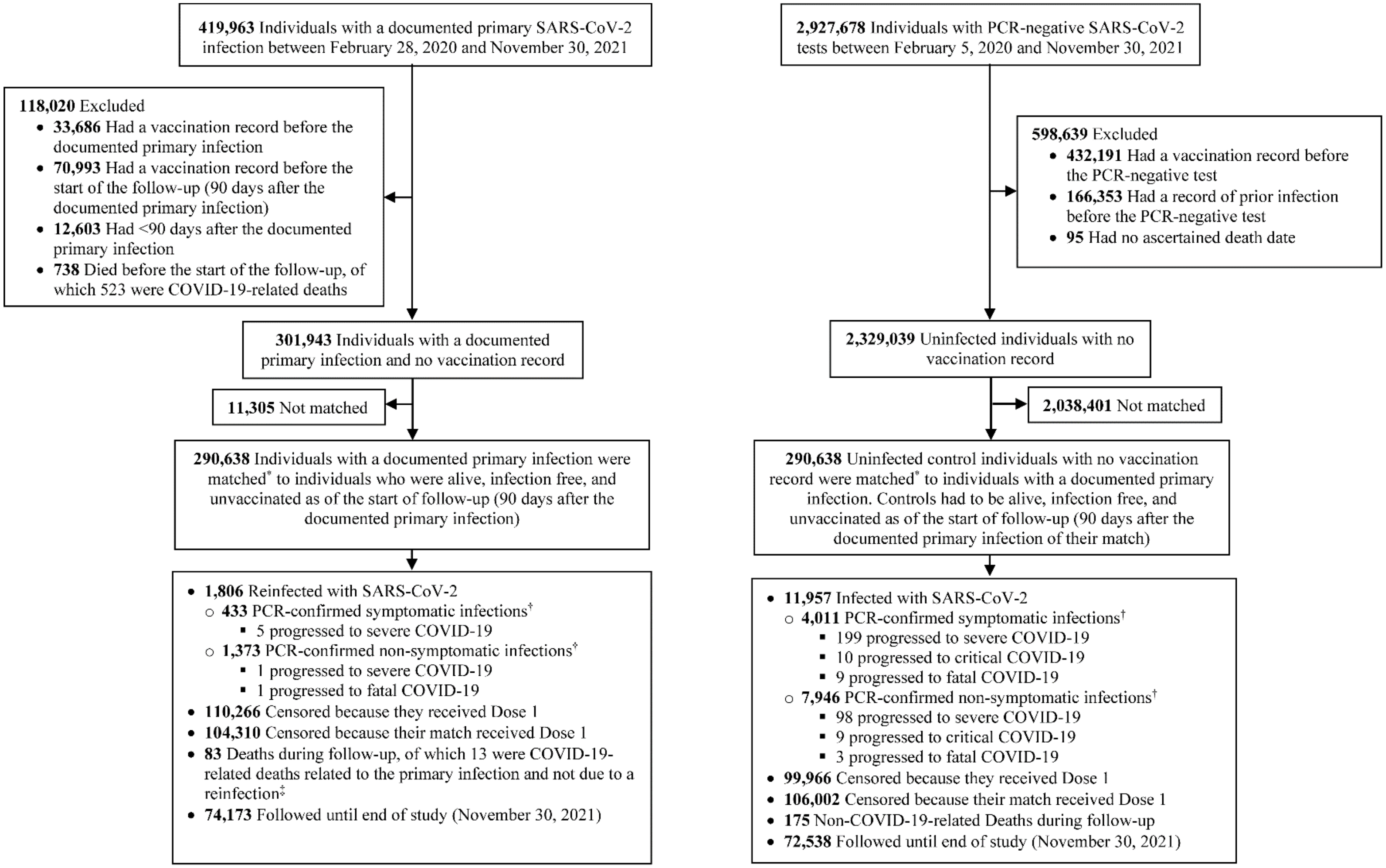

COVID-19 denotes coronavirus disease 2019, PCR polymerase chain reaction, and SARS-CoV-2 severe acute respiratory syndrome coronavirus 2.

*Cohorts were exact-matched in a 1:1 ratio by sex, 10-year age group, nationality, comorbidity count, and calendar week of the SARS-CoV-2 test.

†A PCR-confirmed symptomatic infection was defined as a PCR-positive test conducted because of clinical suspicion due to presence of symptoms compatible with a respiratory tract infection. A PCR-confirmed non-symptomatic infection was defined as an infection diagnosed for a reason for PCR testing other than clinical suspicion. Some of the latter infections developed symptoms after diagnosis and some progressed to severe forms of COVID-19.

‡These deaths occurred among persons who had the death ≥90 days after primary infection but had no record of a documented reinfection during follow-up.

Figure S2. Cohort selection for investigating immune protection of primary infection with a pre-Omicron variant against re-infection with Omicron subvariants (Omicron Reinfection Study).

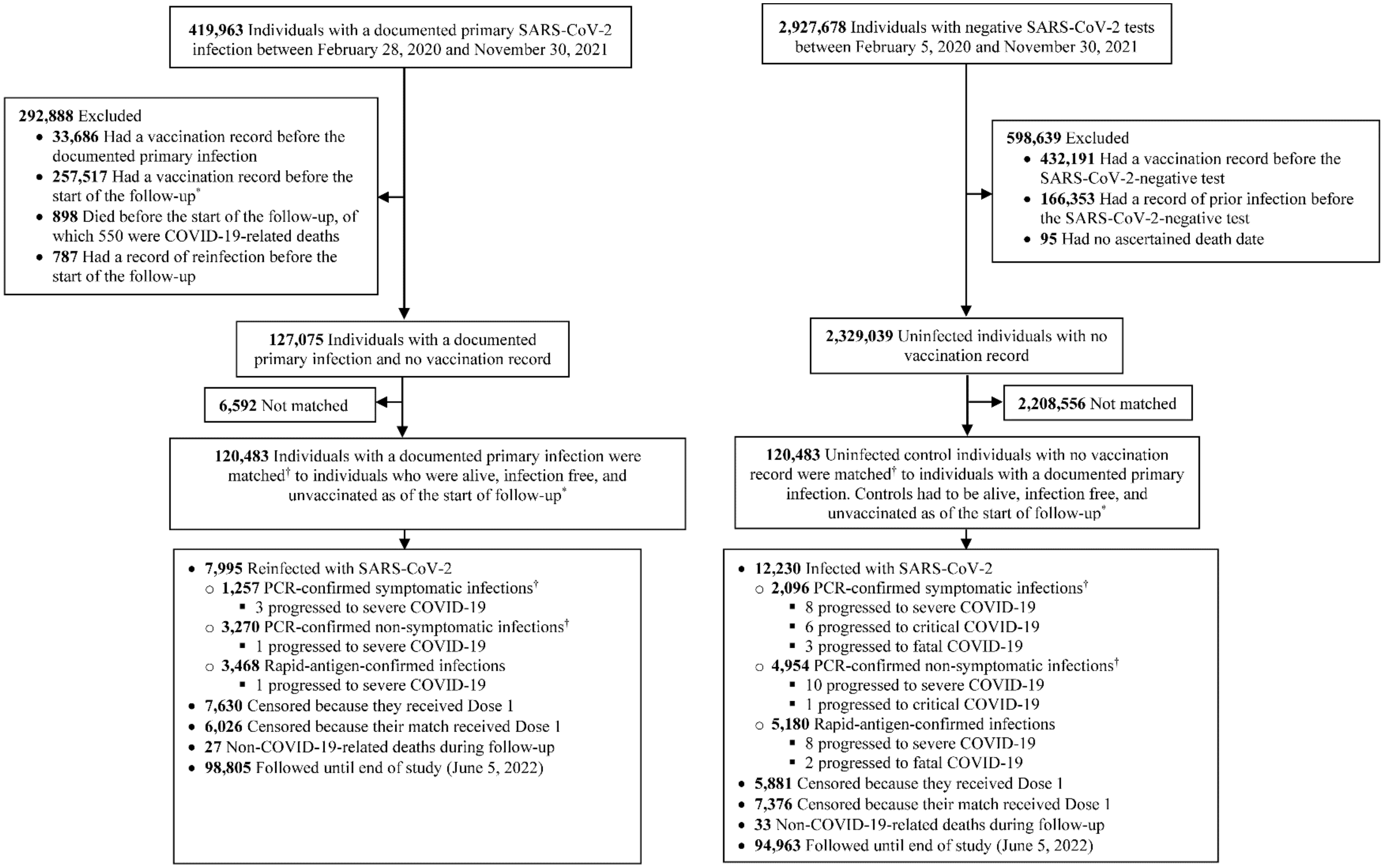

COVID-19 denotes coronavirus disease 2019, PCR polymerase chain reaction, and SARS-CoV-2 severe acute respiratory syndrome coronavirus 2.

[*] Follow-up for cases was from 90 days after the documented primary infection if start of follow-up coincided with the SARS-CoV-2 Omicron wave which started on December 19, 2021, or from December 19, 2021 for earlier infections. Follow-up for controls was from 90 days after the documented primary infection of their match if start of follow-up coincided with the SARS-CoV-2 Omicron wave which started on December 19, 2021, or from December 19, 2021 for earlier infections

[†] Cohorts were exact-matched in a 1:1 ratio by sex, 10-year age group, nationality, comorbidity count, and calendar week of the SARS-CoV-2 test.

[‡] A PCR-confirmed symptomatic infection was defined as a PCR-positive test conducted because of clinical suspicion due to presence of symptoms compatible with a respiratory tract infection. A PCR-confirmed non-symptomatic infection was defined as an infection diagnosed for a reason for PCR testing other than clinical suspicion. Some of the latter infections developed symptoms after diagnosis and some progressed to severe forms of COVID-19. There was no record for reason for testing and presence of symptoms for most of the rapid antigen testing and thus we did not report the symptom status for infections diagnosed using rapid antigen testing.

Figure S3. Cohort selection for investigating immune protection of primary infection with any variant against severe, critical, or fatal COVID-19 due to reinfection with any variant.

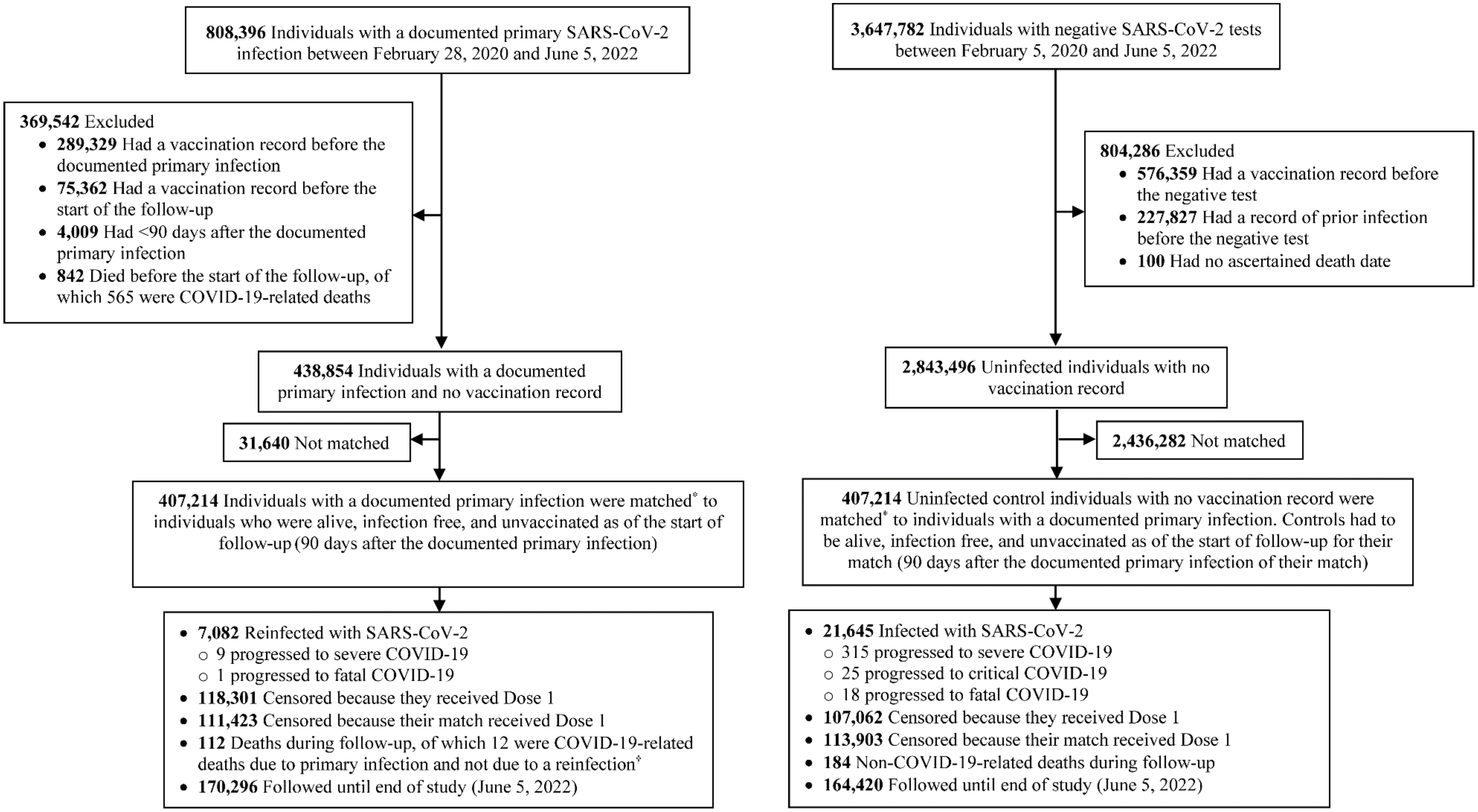

COVID-19 denotes coronavirus disease 2019 and SARS-CoV-2 severe acute respiratory syndrome coronavirus 2.
*Cohorts were exact-matched in a 1:1 ratio by sex, 10-year age group, nationality, comorbidity count, and calendar week of the SARS-CoV-2 test.
†These deaths occurred among persons who had the death ≥90 days after primary infection but had no record of a documented reinfection during follow-up.

Table S2. Baseline characteristics of the eligible and matched primary-infection and infection-naive cohorts in the COVID-19 Severity Reinfection Study.

Characteristics	Full eligible cohorts			Matched cohorts*		
	Primary-infection cohort N=438,854	Infection-naïve cohort N=2,843,496	SMD†	Primary-infection cohort N=407,214	Infection-naïve cohort N=407,214	SMD†
Median age (IQR)—years	30 (16-39)	31 (23-39)	0.15‡	30 (18-38)	30 (19-38)	0.00‡
Age group						
0-9 years	75,287 (17.2)	324,956 (11.4)	0.21	69,059 (17.0)	69,059 (17.0)	0.00
10-19 years	42,913 (9.8)	196,544 (6.9)		36,029 (8.9)	36,029 (8.9)	
20-29 years	97,277 (22.2)	748,686 (26.3)		92,980 (22.8)	92,980 (22.8)	
30-39 years	122,856 (28.0)	880,600 (31.0)		117,269 (28.8)	117,269 (28.8)	
40-49 years	64,930 (14.8)	429,083 (15.1)		60,769 (14.9)	60,769 (14.9)	
50-59 years	25,272 (5.8)	179,111 (6.3)		22,858 (5.6)	22,858 (5.6)	
60-69 years	7,811 (1.8)	64,210 (2.3)		6,492 (1.6)	6,492 (1.6)	
70+ years	2,508 (0.6)	20,306 (0.7)		1,758 (0.4)	1,758 (0.4)	
Sex						
Male	297,317 (67.8)	1,979,916 (69.6)	0.04	280,419 (68.9)	280,419 (68.9)	0.00
Female	141,537 (32.2)	863,580 (30.4)		126,795 (31.1)	126,795 (31.1)	
Nationality§						
Bangladeshi	29,599 (6.7)	175,765 (6.2)	0.26	27,691 (6.8)	27,691 (6.8)	0.00
Egyptian	23,003 (5.2)	136,182 (4.8)		21,534 (5.3)	21,534 (5.3)	
Filipino	32,871 (7.5)	200,002 (7.0)		31,256 (7.7)	31,256 (7.7)	
Indian	108,178 (24.7)	804,623 (28.3)		107,720 (26.5)	107,720 (26.5)	
Nepalese	43,108 (9.8)	253,949 (8.9)		40,098 (9.9)	40,098 (9.9)	
Pakistani	22,647 (5.2)	170,868 (6.0)		21,966 (5.4)	21,966 (5.4)	
Qatari	71,132 (16.2)	249,740 (8.8)		60,438 (14.8)	60,438 (14.8)	
Sri Lankan	11,912 (2.7)	79,189 (2.8)		11,519 (2.8)	11,519 (2.8)	
Sudanese	11,666 (2.7)	62,039 (2.2)		10,914 (2.7)	10,914 (2.7)	
Other nationalities¶	84,738 (19.3)	711,139 (25.0)		74,078 (18.2)	74,078 (18.2)	
Comorbidity count						
None	352,738 (80.4)	2,496,024 (87.8)	0.21	334,409 (82.1)	334,409 (82.1)	0.00
1-2	72,153 (16.4)	277,344 (9.8)		61,720 (15.2)	61,720 (15.2)	
3+	13,963 (3.2)	70,128 (2.5)		11,085 (2.7)	11,085 (2.7)	

IQR denotes interquartile range and SMD standardized mean difference.

*Individuals with a documented primary SARS-CoV-2 infection were exact-matched in a 1:1 ratio by sex, 10-year age group, nationality, comorbidity count, and calendar week of the SARS-CoV-2 test to the first eligible infection-naïve individual.

†SMD is the difference in the mean of a covariate between groups divided by the pooled standard deviation. An SMD <0.1 indicates adequate matching.

‡SMD is for the mean difference between groups divided by the pooled standard deviation.

§Nationalities were chosen to represent the most populous groups in Qatar.

¶These comprise 161 other nationalities in the unmatched primary-infection cohort and 183 other nationalities in the unmatched infection-naïve cohort, and 141 other nationalities in the matched primary-infection cohort and 141 other nationalities in the matched infection-naïve cohort.

References

1. Multiplexed RT-qPCR to screen for SARS-COV-2 B.1.1.7, B.1.351, and P.1 variants of concern V.3. dx.doi.org/10.17504/protocols.io.br9vm966. 2021. (Accessed June 6, 2021, at https://www.protocols.io/view/multiplexed-rt-qpcr-to-screen-for-sars-cov-2-b-1-1-br9vm966.)
2. Abu-Raddad LJ, Chemaitelly H, Butt AA, National Study Group for Covid Vaccination. Effectiveness of the BNT162b2 Covid-19 Vaccine against the B.1.1.7 and B.1.351 Variants. N Engl J Med 2021;385:187-9.
3. Chemaitelly H, Yassine HM, Benslimane FM, et al. mRNA-1273 COVID-19 vaccine effectiveness against the B.1.1.7 and B.1.351 variants and severe COVID-19 disease in Qatar. Nat Med 2021;27:1614-21.
4. Qatar viral genome sequencing data. Data on randomly collected samples. https://www.gisaid.org/phylodynamics/global/nextstrain/. 2021. at https://www.gisaid.org/phylodynamics/global/nextstrain/.)
5. Benslimane FM, Al Khatib HA, Al-Jamal O, et al. One Year of SARS-CoV-2: Genomic Characterization of COVID-19 Outbreak in Qatar. Front Cell Infect Microbiol 2021;11:768883.
6. Hasan MR, Kalikiri MKR, Mirza F, et al. Real-Time SARS-CoV-2 Genotyping by High-Throughput Multiplex PCR Reveals the Epidemiology of the Variants of Concern in Qatar. Int J Infect Dis 2021;112:52-4.
7. Chemaitelly H, Tang P, Hasan MR, et al. Waning of BNT162b2 Vaccine Protection against SARS-CoV-2 Infection in Qatar. N Engl J Med 2021;385:e83.
8. Saththasivam J, El-Malah SS, Gomez TA, et al. COVID-19 (SARS-CoV-2) outbreak monitoring using wastewater-based epidemiology in Qatar. Sci Total Environ 2021;774:145608.
9. Abu-Raddad LJ, Chemaitelly H, Ayoub HH, et al. Effect of mRNA Vaccine Boosters against SARS-CoV-2 Omicron Infection in Qatar. N Engl J Med 2022;386:1804-16.
10. Tang P, Hasan MR, Chemaitelly H, et al. BNT162b2 and mRNA-1273 COVID-19 vaccine effectiveness against the SARS-CoV-2 Delta variant in Qatar. Nat Med 2021;27:2136-43.
11. Altarawneh HN, Chemaitelly H, Ayoub HH, et al. Effects of Previous Infection and Vaccination on Symptomatic Omicron Infections. N Engl J Med 2022;387:21-34.
12. Altarawneh HN, Chemaitelly H, Hasan MR, et al. Protection against the Omicron Variant from Previous SARS-CoV-2 Infection. N Engl J Med 2022;386:1288-90.
13. Chemaitelly H, Ayoub HH, AlMukdad S, et al. Duration of mRNA vaccine protection against SARS-CoV-2 Omicron BA.1 and BA.2 subvariants in Qatar. Nat Commun 2022;13:3082.
14. Qassim SH, Chemaitelly H, Ayoub HH, et al. Effects of BA.1/BA.2 subvariant, vaccination, and prior infection on infectiousness of SARS-CoV-2 omicron infections. J Travel Med 2022.
15. World Health Organization. COVID-19 clinical management: living guidance. Available from: https://www.who.int/publications/i/item/WHO-2019-nCoV-clinical-2021-1. Accessed on: May 15, 2021. 2021.
16. World Health Organization. International guidelines for certification and classification (coding) of COVID-19 as cause of death. Available from: https://www.who.int/classifications/icd/Guidelines_Cause_of_Death_COVID-19-20200420-EN.pdf?ua=1. Document Number: WHO/HQ/DDI/DNA/CAT. Accessed on May 15, 2021. 2020.
17. Nishiura H, Schwehm M, Eichner M. Still protected against smallpox? Estimation of the duration of vaccine-induced immunity against smallpox. Epidemiology 2006;17:576-81.
18. Abu-Raddad LJ, Chemaitelly H, Malek JA, et al. Assessment of the Risk of Severe Acute Respiratory Syndrome Coronavirus 2 (SARS-CoV-2) Reinfection in an Intense Reexposure Setting. Clin Infect Dis 2021;73:e1830-e40.

19. Abu-Raddad LJ, Chemaitelly H, Malek JA, et al. Two prolonged viremic SARS-CoV-2 infections with conserved viral genome for two months. Infect Genet Evol 2021;88:104684.
20. Hansen CH, Michlmayr D, Gubbels SM, Molbak K, Ethelberg S. Assessment of protection against reinfection with SARS-CoV-2 among 4 million PCR-tested individuals in Denmark in 2020: a population-level observational study. Lancet 2021;397:1204-12.
21. Abu-Raddad LJ, Chemaitelly H, Coyle P, et al. SARS-CoV-2 antibody-positivity protects against reinfection for at least seven months with 95% efficacy. EClinicalMedicine 2021;35:100861.
22. Kojima N, Shrestha NK, Klausner JD. A Systematic Review of the Protective Effect of Prior SARS-CoV-2 Infection on Repeat Infection. Eval Health Prof 2021;44:327-32.
23. Pilz S, Theiler-Schwetz V, Trummer C, Krause R, Ioannidis JPA. SARS-CoV-2 reinfections: Overview of efficacy and duration of natural and hybrid immunity. Environ Res 2022:112911.
24. Abu-Raddad LJ, Chemaitelly H, Ayoub HH, et al. Characterizing the Qatar advanced-phase SARS-CoV-2 epidemic. Sci Rep 2021;11:6233.
25. Ayoub HH, Chemaitelly H, Seedat S, et al. Mathematical modeling of the SARS-CoV-2 epidemic in Qatar and its impact on the national response to COVID-19. J Glob Health 2021;11:05005.
26. Coyle PV, Chemaitelly H, Ben Hadj Kacem MA, et al. SARS-CoV-2 seroprevalence in the urban population of Qatar: An analysis of antibody testing on a sample of 112,941 individuals. iScience 2021;24:102646.
27. Al-Thani MH, Farag E, Bertollini R, et al. SARS-CoV-2 Infection Is at Herd Immunity in the Majority Segment of the Population of Qatar. Open Forum Infect Dis 2021;8:ofab221.
28. Jeremijenko A, Chemaitelly H, Ayoub HH, et al. Herd Immunity against Severe Acute Respiratory Syndrome Coronavirus 2 Infection in 10 Communities, Qatar. Emerg Infect Dis 2021;27:1343-52.
29. Abu-Raddad LJ, Dargham S, Chemaitelly H, et al. COVID-19 risk score as a public health tool to guide targeted testing: A demonstration study in Qatar. medRxiv 2021:2021.03.06.21252601.
30. Abu-Raddad LJ, Chemaitelly H, Ayoub HH, et al. Relative infectiousness of SARS-CoV-2 vaccine breakthrough infections, reinfections, and primary infections. Nat Commun 2022;13:532.
31. Abu-Raddad LJ, Chemaitelly H, Bertollini R, National Study Group for Covid Epidemiology. Severity of SARS-CoV-2 Reinfections as Compared with Primary Infections. N Engl J Med 2021;385:2487-9.
32. Abu-Raddad LJ, Chemaitelly H, Ayoub HH, et al. Introduction and expansion of the SARS-CoV-2 B.1.1.7 variant and reinfections in Qatar: A nationally representative cohort study. PLoS Med 2021;18:e1003879.
33. Chemaitelly H, Bertollini R, Abu-Raddad LJ, National Study Group for Covid Epidemiology. Efficacy of Natural Immunity against SARS-CoV-2 Reinfection with the Beta Variant. N Engl J Med 2021;385:2585-6.
